# Biosensors for D-Amino Acids: Detection Methods and Applications

**DOI:** 10.3390/ijms21134574

**Published:** 2020-06-27

**Authors:** Elena Rosini, Paola D’Antona, Loredano Pollegioni

**Affiliations:** Department of Biotechnology and Life Sciences, University of Insubria, via J.H. Dunant 3, 21100 Varese, Italy; p.dantona@uninsubria.it (P.D.); loredano.pollegioni@uninsubria.it (L.P.)

**Keywords:** D-amino acids, biosensors, human health, food quality, neurotransmission, biomarkers

## Abstract

D-enantiomers of amino acids (D-AAs) are only present in low amounts in nature, frequently at trace levels, and for this reason, their biological function was undervalued for a long time. In the past 25 years, the improvements in analytical methods, such as gas chromatography, HPLC, and capillary electrophoresis, allowed to detect D-AAs in foodstuffs and biological samples and to attribute them specific biological functions in mammals. These methods are time-consuming, expensive, and not suitable for online application; however, life science investigations and industrial applications require rapid and selective determination of D-AAs, as only biosensors can offer. In the present review, we provide a status update concerning biosensors for detecting and quantifying D-AAs and their applications for safety and quality of foods, human health, and neurological research. The review reports the main challenges in the field, such as selectivity, in order to distinguish the different D-AAs present in a solution, the simultaneous assay of both L- and D-AAs, the production of implantable devices, and surface-scanning biosensors. These innovative tools will push future research aimed at investigating the neurological role of D-AAs, a vibrant field that is growing at an accelerating pace.

## 1. Introduction

α-Amino acids (AAs) are constituted of a central carbon (the so-called α-carbon) linked to four different substituents: a hydrogen, a carboxyl group, an amine group, and a side chain (which distinguishes each AA). With the only exception of glycine, the α-carbon is linked to four different groups; thus, it represents a chiral center and generates two molecules that are mirror images of each other: these enantiomers are termed levorotatory (L-) and dextrorotatory (D-) AAs. In large part, biomolecules are constituted of L-AAs (such as proteins) and the presence of D-AAs was thus undervalued [1]. In the past 25 years, a number of investigations highlighted different and specific roles of D-AAs in living organisms, as reviewed in [1,2,3,4]. In this context, the rapid and selective determination of D-AAs (and their derivatives) in biological matrices is expected to strongly impact both life sciences and industrial sectors. D-AAs can be detected by different techniques (especially gas chromatography, reversed-phase HPLC, and capillary electrophoresis): these methods show various disadvantages, such as the cost and time for each analysis and the impossibility to use in online applications [5,6,7,8,9,10,11]. The use of biosensors overcomes these drawbacks and offers intriguing potential, in terms of simplicity and speed of operation, high specificity, cost effectiveness, user-friendliness, integration into portable devices, etc. [12,13].

In the present review, we provide a status update concerning biosensors for detecting and quantifying D-AAs and their applications for safety and quality of foods, human health, and neurological research. The review concludes with a focus on the main challenges for producing devices able to push the field and develop novel scientific and industrial applications.

## 2. Biosensors

The term “biosensor” is defined by the International Union of Pure and Applied Chemistry as “a device that uses specific biochemical reactions mediated by isolated enzymes, immunosystems, tissues, organelles or whole cells to detect chemical compounds, usually by electrical, thermal or optical signals” [14,15,16]. Accordingly, a biosensor consists of three main components: (i) bioreceptor: a molecule (i.e., enzyme, cell, or antibody) that recognizes the analyte; (ii) transducer: an element that converts the biorecognition event into a measurable signal; and (iii) electronics: a signal-processing system to convert the transduced signal in display form, depicting the presence of the target analyte.

**Figure 1:**
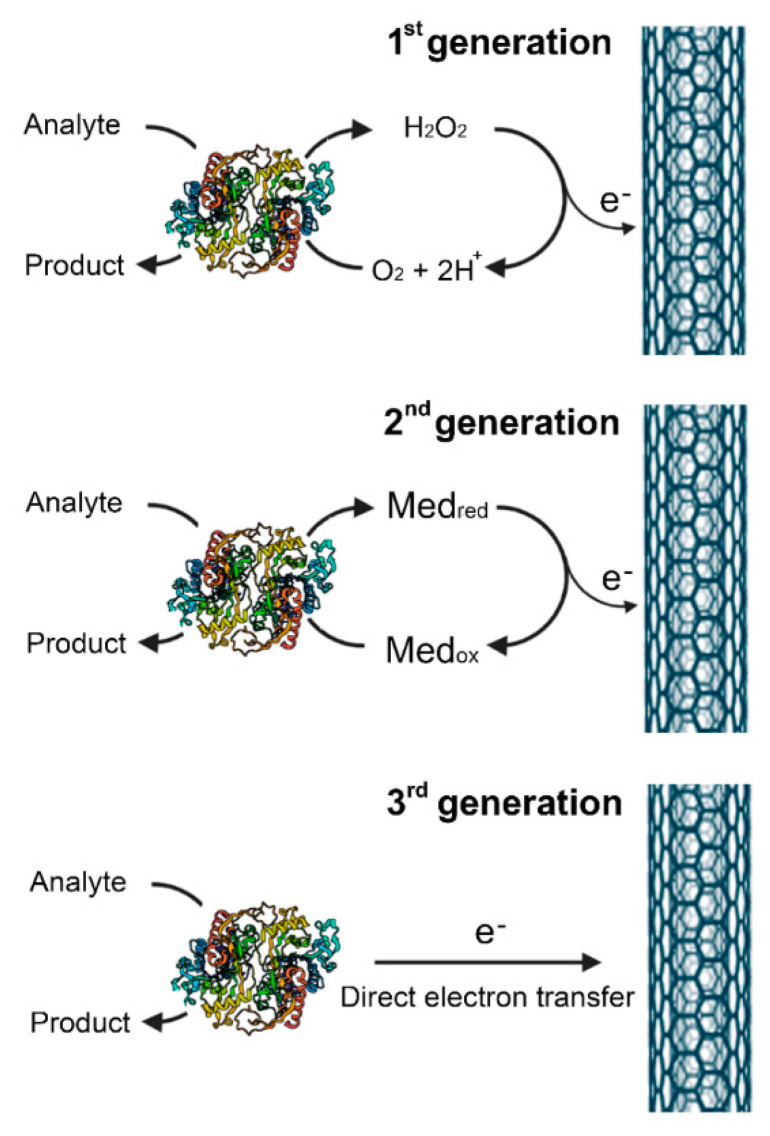
Different generations of amperometric biosensors. In the first biosensor generation, the decrease in oxygen concentration or the produced hydrogen peroxide are measured; in the second biosensor generation, specific mediators transfer the electrons to the electrode; in the third biosensor generation, the electrons are directly transferred from the enzyme to the electrode.

Based on the working principle, biosensors are classified into different types. The physical change produced by the biorecognition event (the signal) may be: (i) heat output (calorimetric biosensors); (ii) a different charge distribution (potentiometric biosensors); (iii) the movement of electrons produced in a redox reaction (amperometric biosensors); (iv) an absorbance/fluorescence change (optical biosensors); or (v) an oscillation change due to mass binding on a crystal surface (piezo-electric biosensors). Amperometry is the most commonly reported method among electrochemical biosensors: a constant potential is applied between a working and a reference electrode, and the imposed potential encourages redox reactions to take place, yielding a net current proportional to the concentration of analyte in solution [17,18,19]. Typically, oxidases have been the most frequently exploited enzymes for these biosensors: the oxygen consumed or the hydrogen peroxide generated is monitored [20,21,22,23]. Three types of amperometric biosensors have been developed [18,24]. In the first biosensor generation, the reaction product diffuses to the transducer to give the electrical signal; the second generation involves specific mediators (i.e., organic compounds that transfer the electrons directly to the electrode, overcoming the dependence on the dissolved oxygen concentration); and in the third biosensor generation, the reaction itself causes the electrical response (Figure 1). Fluorescence-based biosensors are also frequently used because of their high sensitivity: the analytical signal arises from a fluorescence or phosphorescence emission process.

Biosensors can operate as offline devices (i.e., target analytes are measured after sample recovery), online devices (i.e., real-time measurement by a device inserted into the body or the production line), and in vivo sensors (i.e., continuous monitoring of the analyte concentration by a device implanted into the body) [18,25]. 

Biosensors have gained considerable attention in recent years in medical diagnostics applications and in a number of other sectors, such as the pharmaceutical, food, and agricultural industries [13,17]. However, medical applications dominate the field: indeed, disposable blood glucose biosensors account for 85% of the current total biosensors market [13,23]. Clinically, biosensors are widely and successfully employed to detect disease-specific biomarkers: health status, onset of disease, and disease progression can be detected, helping to assess medical treatments [13,26]. The advantages of biosensors have encouraged researchers to develop new technologies and the industry is now worth billions of dollars, the major impetus coming from the healthcare industry [13,17]. Biosensors represent a rapidly expanding field, with a global market size of 19.6 billion of dollars in 2019 and anticipated to reach 36 billion of dollars by 2027, with a compound annual growth rate of 9% [27].

## 3. D-Amino Acids

For a long time, D-AAs were considered to be of bacterial origin only since they are peptidoglycan components of the bacterial cell wall and contribute to making it more resistant to proteases and to some antibiotics [28]. D-AAs in the bacterial cell wall also play a role in bacterial selection: the peptidoglycan is a dynamic structure which is modified by introducing alternative D-AAs during the stationary phase [29,30]. D-AAs have also been identified in biologically active peptides (homologous or identical to mammalian hormones and/or neurotransmitters) secreted by amphibians, and in the cellular fluids of some marine worms and invertebrates [4]. In addition, D-AAs are present in foodstuffs, both naturally (such as in fruit juices and pulp, cereals, milk, etc.) or generated by processing treatments aimed to improve flavor (such as cheese fermentation and ripening), consistency, and nonperishability, or due to adulteration (hydrolyzed proteins have been added to foods to cover low nutrient content); see [10].

The presence of D-AAs in animals was definitively established in 1969 [31]: they were assumed to originate from endogenous microbial flora, from diet, or from spontaneous racemization of L-AAs. Then, 23 years later, a specific biological function in mammals was attributed to selected D-AAs when Hashimoto et al. reported relevant concentrations of D-Asp and D-Ser in the central nervous system [32] and when D-Ser was demonstrated to be produced in the brain by a specific racemase [33]. D-Ser is the main coagonist of N-methyl-D-aspartate (NMDA) receptors and D-Asp is an agonist of the same receptors. These D-AAs affect neurotransmission and main brain functions, such as learning and memory [34,35,36]. In addition, D-Asp acts on further receptors and is involved in the synthesis of different hormones and of melatonin [37,38,39]. Altered levels of various D-AAs have been related to different pathological states, such as chronic kidney diseases [40,41] and neurological disorders, i.e., schizophrenia, Alzheimer’s disease, etc. [42,43,44,45,46,47]. Accordingly, the determination of D-AAs levels in biological fluids and tissues is highly relevant to human health.

The D-AAs concentration and composition in food samples are considered an index for food quality assessment, a marker for food ripening, and a means of identifying microbiological contamination [10,48]. D-AAs are valuable pharmaceuticals in their own right and represent building blocks for numerous drugs: an assessment of their concentration and purity degree is frequently required [1,3,49].

D-AAs can be determined using chiral HPLC, gas chromatography, and capillary electrophoresis coupled with different detection systems [4,11,50]. The main drawbacks of these techniques are: (i) the time required for each measurement; (ii) the need for expensive instrumentation and highly skilled operators; and (iii) the inability to be directly employed to analyze complex samples (e.g., food samples and tissue extracts). Enzymatic assays exploiting the strict enantioselectivity of selected enzymes and characterized by fast, easy, and inexpensive procedures that have also been set up. The flavoenzyme D-amino acid oxidase (DAAO, EC 1.4.3.3), which catalyzes the oxygen-dependent deamination of D-AAs into the corresponding α-keto acids, ammonia, and hydrogen peroxide [51], has been used to detect D-AAs [52]. Over the years, the substrate specificity of the enzyme was enlarged by protein engineering studies generating a number of variants active on unnatural AAs [53,54,55], selective for few D-AAs [54,56], or with a higher oxygen reactivity [57,58]. The main disadvantages of enzymatic assays are [52]: (i) the presence of compounds in the sample that alter the detected signal (especially when the absorbance in the UV region is used, i.e., for the direct determination of α-keto acids) or affect the enzymatic activity; (ii) the low sensitivity of the direct assay of α-keto acids, due to low extinction coefficients; (iii) the response variability of the most sensitive fluorescence-based assays; and (iv) the need for calibration curves and thus for trained personnel.

Biosensors represent a suitable alternative here: the possibility of employing engineered DAAO variants coupled to novel transduction systems makes it possible to propose “the latest-generation biosensors” as suitable analytical tools for determining D-AAs levels even from complex matrices. Main benefits are: operational simplicity and speed, high specificity, very low reagent consumption, cost effectiveness, user-friendliness, and integration into a small portable device for determining multiple parameters and for continuous monitoring [12,13].

## 4. Detection of D-AAs in Foods by Electrochemical Biosensors

The D-AAs content in the foods we eat is determined by the raw material, the production methods, and the microbial contaminations. Thus, D-AAs detection can represent a useful marker of quality, authenticity, and safety of foods. A number of biosensors for simple, rapid, and specific detection of D-AAs have been reported, mainly based on the use of DAAO from different sources and applying several immobilization methods.

The flow electrochemical biosensor based on the stereoselective DAAO from *Rhodotorula gracilis* (RgDAAO) adsorbed on a graphite electrode represented such a pioneering device: at the working potential of +400 mV, it showed a response time of approximately 5 min, a linear response between 0.2–3 mM D-Ala, a limit of detection (LOD) value of 0.15 mM, and good reproducibility [21]; see Table 1. This disposable device required a very low amount of enzyme (10 µg) for each determination, with no need to supplement exogenous flavin adenine dinucleotide (FAD). The biosensor has been used to assay D-AAs content in milk samples stored at 4 °C for 1 month: the results were in good agreement with the values obtained by HPLC analyses.

Later on, biosensors for monitoring fermentation processes were developed using the immobilized DAAO from pig kidney (pkDAAO) in a flow-injection analysis (FIA) system. The enzyme solution was immobilized with glutaraldehyde on a small pig membrane in a thin-layer Plexi-cell [59]. The H_2_O_2_ produced during the enzymatic reaction was measured by an amperometric detector at a fixed +100 mV potential. The biosensor showed an LOD value of 0.2 mM and good reproducibility (for 25 sequential measurements) and was used for more than 1000 measurements, making it possible to analyze 50–60 samples per hour (Table 1). The optimized biosensor was applied to monitor different stages of the fermentation process of ginger and brown beers. Determining D-Ala to control fermentation processes was also carried out by a similar FIA system consisting of a pkDAAO reactor and a pyruvate oxidase (PO, EC 1.2.3.3) electrode in the flow cell [60]: pyruvic acid generated by pkDAAO starting from D-Ala was further oxidized by PO. pkDAAO and catalase (added to eliminate hydrogen peroxide) were coimmobilized on long-chain amino-alkyl controlled pore glass, activated with glutaraldehyde, and packed into a polypropylene reactor tube; the PO-immobilized membrane was placed on a Teflon membrane in an oxygen electrode. This enzymatic system, to which 10 µM FAD and 1 mM thiamine pyrophosphate were added as cofactors (a main drawback in utilization), showed good repeatability up to 60 measurements (each assay requiring 12 min for completion), with an LOD value of 0.05 mM (Table 1). The results obtained for fish sauces were in good agreement with those determined by chromatographic methods.

The immobilization of DAAO on a graphite working electrode of modified screen-printed sensors to facilitate the H_2_O_2_ oxidation at a decreased operating potential has also been reported. pkDAAO was immobilized on an electrocatalytic rhodinized carbon: the device responded within 4 min to five common D-AAs (i.e., D-Ala, D-Arg, D-Met, D-Phe, and D-Val), with good reproducibility and stability over 56 days; see Table 1 [61]. The device was successfully used as a disposable apparatus, thus minimizing the electrode fouling effects, to monitor the D-AA content in aged milk samples. A similar device was generated based on immobilized pkDAAO on a screen-printed strip modified by adding a layer of Prussian Blue as a mediator and covered by a layer of Nafion [62]. The H_2_O_2_ produced by the DAAO reaction oxidized the reduced form of the mediator, where electron transfer was detected amperometrically at a -50 mV potential, representing an example of second-generation amperometric biosensors; see Figure 1. All measurements were carried out in the presence of 10 µM FAD and the response usually stabilized within 3 min. The optimized biosensor showed an LOD value of 1 µM and a long-term stability up to 10 days: it was used to assay the D-Ala content in several commercial fruit juices and milk samples.

**Figure 2:**
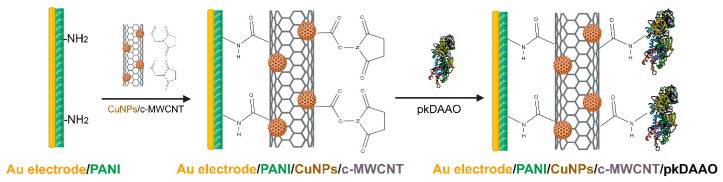
Amperometric biosensor for detection of D-AAs in foodstuffs. At first, multiwall carbon nanotubes (MWCNTs) decorated with copper nanoparticles (CuNPs) were electrodeposited on a gold electrode covered by the conducting polymer polyaniline (PANI); then, the transducer enzyme pkDAAO was covalently immobilized. The sensor was operated at a working potential of +90 mV. Modified from [63].

The use of nanomaterials to construct enzyme electrodes has produced higher stability and sensitivity. Goat kidney DAAO was immobilized onto zinc sulfide nanoparticles (ZnSNPs) covered by a protective layer of polyindole-5-carboxylic acid film and electrodeposited on a gold electrode [64]. The biosensor showed a rapid response within 3 s, at a fixed potential of +150 mV, with an LOD value of 1 µM (Table 1). The biosensor retained about 80% of its initial response after 120 uses over a period of 60 days. The same group designed an improved biosensor by covalently immobilizing pkDAAO onto multiwall carbon nanotubes (MWCNTs) decorated with copper nanoparticles (CuNPs) and electrodeposited on the surface of a gold electrode along with the conducting polymer polyaniline (PANI); see Figure 2 [63]. Owing to the synergistic influence of these three components a very rapid response was achieved within 2 s, with LOD and limit of quantification (LOQ) values of 0.2 µM and 0.03 µM, respectively (Table 1). The biosensor maintained 70% of its initial response when used 150 times over a period of 140 days. A negligible interference of uric acid, ascorbic acid, glycine, and glucose was apparent, due to the low working potential applied (+90 mV). Both of these biosensors were used to assay D-AAs levels in fruit juices, showing a high accuracy when compared to a standard colorimetric assay.

A significant challenge was overcome by producing a device in which the D-AAs’ composition of assayed samples did not alter the response and was thus useful for determining the total D-AAs content. In this context, the T60A/Q144R/K152E and M213E variants of RgDAAO covalently immobilized on an Amberzyme Oxirane support, which showed the lowest variability in response as a function of D-AAs composition, particularly when the D-Ala concentration accounted for ≥20% of the mixture composition [22]. The amount of D-AAs determined in Grana Padano cheese samples was in good agreement with the value obtained by classical chromatographic procedures but the result was delivered in only a few seconds and by using a simple method. In the case of cheese, this device can follow the ripening process, while for other foodstuffs (milk, baby food), it represents a biomarker of microbial contamination regardless of the bacteria (different bacteria differ in D-AAs composition of the cell wall; see paragraph 3 above). 

## 5. Detection of D-AAs for Biomedical Applications

Biosensors have gained considerable attention in medical diagnostics to detect a wide range of molecules by means of multiplex analyses. Analytical properties and performances of D-AAs selective biosensors for biomedical applications (most frequently amperometric biosensors), reporting the validation on biological samples, are compared and summarized in Table 2.

### 5.1. Enzymatic Biosensors

Of main relevance are the enzyme-based electrochemical sensors because of advantages such as operational simplicity, low cost, high selectivity, and suitability for real-time detection. In this context, the use of conducting polymers was investigated: DAAO was entrapped by electropolymerization and the polymeric film covered uniformly the surface of the working electrode. As an example, an amperometric biosensor was constructed by immobilizing DAAO from goat kidney on a hybrid film of nickel hexacyanoferrate polypyrrole deposited over the surface of a glass covered carbon electrode [65]. This modified electrode was used to detect D-Ala in serum and urine samples of healthy individuals and patients with kidney disorders. The biosensor showed an optimum response within 1 s, exhibiting an excellent sensitivity with an LOD value of 1.5 µM (Table 2). The enzyme electrode was used more than 50 times over 2 months, showing a 98.8% analytical recovery of 10 mM D-Ala when added to serum samples.

Carbon nanotubes (CNTs) provide excellent electroconductivity and are thus well suited to act as a scaffold for enzyme immobilization and to enhance electron transfer to the electrodes. Using an electrochemical graphene nanoribbon-based biosensor, obtained by chemical oxidation of CNTs, D-Met and D-Tyr, considered biomarkers for bacterial diseases related to *Vibrio cholera* and *Bacillus subtilis*, respectively, could be rapidly, selectively, accurately, and reproducibly determined [66]. Both D-AAs were detected in urine samples with a response time of 360 s, an excellent linearity, and an LOD value of 60 μM for D-Tyr (Table 2).

A selective and sensitive voltammetric bi-enzyme biosensor to detect D-Ala was fabricated by simultaneously and electrostatically immobilizing pkDAAO and hemoglobin on MnO_2_-NPs covered with polythiophene (PTh) [70]. Hb was used to improve oxygen concentrations locally since it is a cosubstrate of DAAO that is frequently present at a limiting level. The biosensor DAAO-Hb/MnO_2_-NPs/PTh showed a linear response in the 0.04 to 9.0 μM D-Ala concentration range, with an LOD of 41 nM (Table 2). The proposed biosensor was applied successfully to human serum as a real sample, exhibiting a rapid response and a long-term stability (up to 42 days). A similar bi-enzyme biosensor was previously developed by immobilizing pkDAAO and horseradish peroxidase on a pentacyanoferrate-bound poly(1-vinylimidazole) polymer as mediator and covering it with a Nafion film [71]. This polymer is an electroconducting hydrogel that can covalently bind enzymes through poly(ethyleneglycol)diglycidyl ether and favors shuttle electrons between enzyme and electrode. The biosensor responded to D-Ala and D-Ser within 50 s, showing a linear response in the 10 to 350 μM and 5 to 150 μM concentration range, respectively, with an LOD value of 2 μM for both D-AAs (Table 2).

A sol-gel sensor technology was applied to manufacture an enzymatic biosensor based on the immobilization of pkDAAO by sol-gel on a glass covered carbon electrode surface modified by electrodeposition of gold nanofilm (Au-NF) and MWCNTs [68]. The linear relationship between biosensor response and D-Ala concentration was excellent (ranging from 0.25 to 4.5 μM). The biosensor exhibited an LOD value of 20 nM, quick response, good stability (up to 4 weeks), and high analytical recovery (98.9%) of D-Ala added to human serum (Table 2).

**Figure 3:**
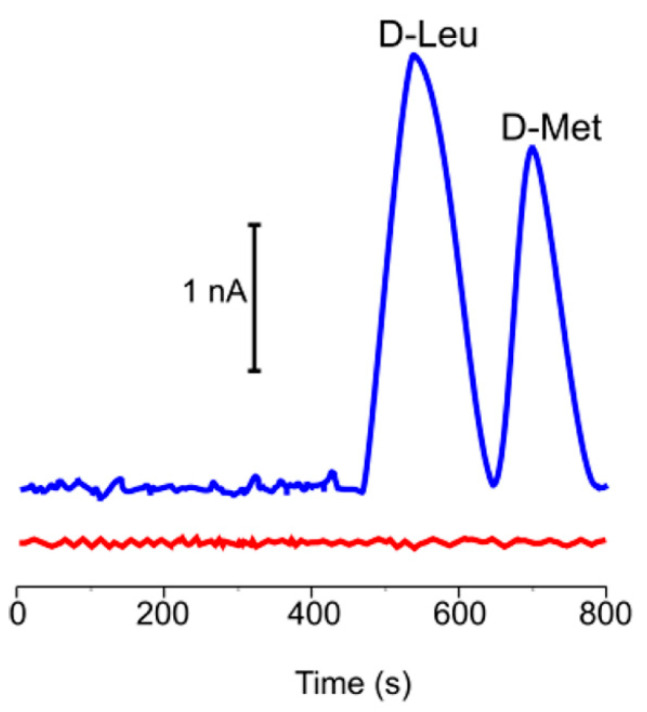
Microchip electropherograms of DAAO (0.5 mg/mL) reaction with a mixture of D-Leu and D-Met (blue line) and L-Leu and L-Met (red line). This figure is reprinted with permission from https://pubs.acs.org/doi/10.1021/acs.analchem.5b00979 [67]. Further permissions related to the material excerpted should be directed to the ACS.

An innovative biosensor for both the racemic resolution and detection of D-Met and D-Leu (considered biomarkers of cholera disease) was based on an electrochemical microfluidic chip [67]. Hybrid polymer/graphene-based electrodes were end-channel coupled to a microfluidic system: DAAO was used to direct both the D-AAs separation and the reaction with each separated D-AA, thus avoiding any covalent immobilization of the enzyme. D-Leu and D-Met were isolated as well-defined peaks (see Figure 3), using a low amount of biological sample and with an LOD value of 1 mM (Table 2). This portable device was proposed for bacterial screening in biological samples in the future.

An original strategy for detecting biomarkers “on-the-fly” relies on self-propelled enzyme motion. Conical motors only millimeters in size containing pkDAAO and sodium dodecylsulfate are propelled by the Marangoni effect, due to the asymmetric release of surfactant by the sharp end, based on surface tension gradient [69]. The continuous release of fresh DAAO induced by the self-propelled movement resulted in a rapid enzymatic reaction without the need for external stirring or chemical and physical adhesion of the enzyme. Ultra-fast detection (<2 min), excellent linearity in the 0.007 to 3.33 mM D-Val concentration range, and an LOD value of 6 μM (Table 2) highlighted the potential application for relevant clinical diseases such as phenylketonuria and *V. cholera* infections.

### 5.2. Microbiosensors

Recently, researchers have developed various innovative strategies to miniaturize devices so that they can be used as an integral part of tissue-engineering systems and implanted in vivo. Noteworthy is an amperometric biosensor based on a platinum wire microbiosensor (25 × 150 µm) and covered with a membrane of poly-*m*-phenylenediamine (PPD) that was implanted in animals to monitor D-Ser levels in the central nervous system [72,79]. The microbiosensor showed an LOD value of 16 nM and a response time within 2 s (Table 2). This device was then optimized in terms of stability and sensitivity by immobilizing RgDAAO using poly(ethyleneglycol)diglycidyl ether [73]. This microbiosensor was widely used over the years to investigate the role of D-Ser in the brain under physiological and pathological conditions [72,80,81,82,83].

**Figure 4:**
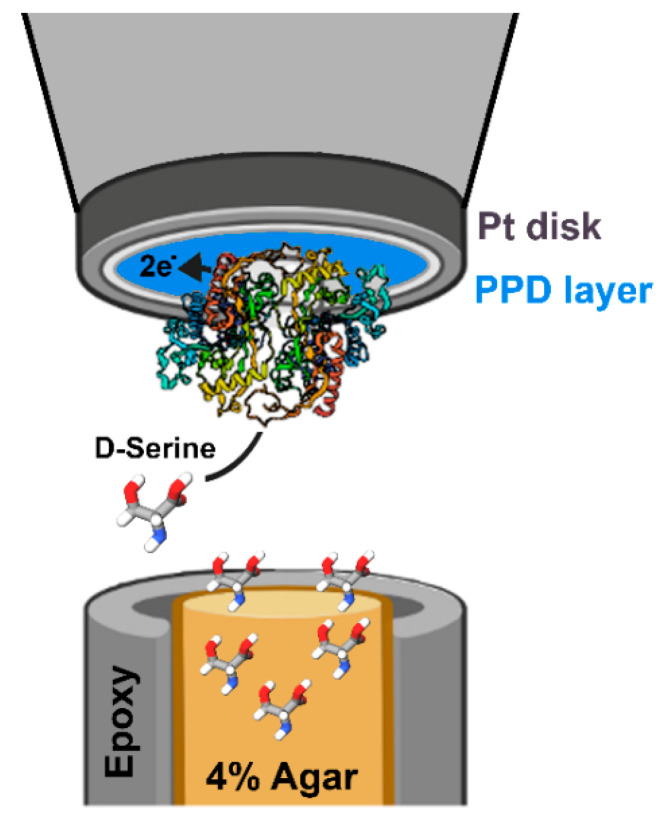
Set up of a scanning electrochemical microscopy (SECM) system for imaging surface topography and local reactivity by scanning a small-scale probe (in the micrometer range) over an immersed substrate immersed in 4% agar and embedded in an epoxy puck, while recording the current. The system is based on a disk-shaped amperometric RgDAAO biosensor deposited on a 25 μm diameter platinum disk microelectrode with an electrodeposited PPD layer. Modified from [84].

Later on, a disk-shaped amperometric RgDAAO biosensor was manufactured based on a platinum disk microelectrode, 25 μm in diameter, with an electrodeposited PPD layer: this biosensor was 1−5 orders of magnitude smaller than previously reported probes and showed a detection limit of 0.6 μM (Table 2) [74]. This apparatus was applied for measuring the release of D-Ser in the brain of *Xenopus laevis* tadpoles in vivo, a model system used to investigate synaptic transmission and plasticity under physiological and pathological conditions. An optimized version of such a biosensor was used in a scanning electrochemical microscopy (SECM, a high-resolution electroanalytical technique for imaging surface topography, Figure 4) for the detection of D-Ser release [84]. SECM records a current during the scanning of a surface using a small-scale probe (in the micrometer range) over an immersed substrate. This technique could be useful to investigate the role of D-Ser in brain development and diseases by mapping its local release from astrocytes and neurons.

RgDAAO was also used as bioreceptor for an amperometric sensor for D-Ser with self-referencing ceramic-based microelectrode arrays [75]. This system used two channels, each consisting of a pair of platinum recording sites named D-AAs detecting channels and sentinel channels, which are spaced only tens of micrometers apart. Both channel types measured background noise and interference activity, but only the enzyme-coated sites (the D-AAs detecting channels) were able to detect D-Ser. With this set up, background noise and neurochemical interferent activity could easily be subtracted and reached an LOD of 0.17 µM (Table 2): the device could be used for freely moving studies.

### 5.3. Fluorescence Biosensors

Recently, fluorescence-based nanobiosensors were developed for several medical applications: this detection system is sophisticated and expensive, thus making it difficult to design a portable device for in situ monitoring of D-AAs.

An earlier chemiluminescence flow biosensor was developed using a column with immobilized pkDAAO onto amine-modified silica gel: hydrogen peroxide produced by the enzymatic reaction on total D-AAs was reacted with luminol in the presence of ferricyanide (detection limit was 0.45 µM) [85]. Later on, the pyridoxal 5’-phosphate containing enzyme D-serine dehydratase has been used for implementing a biosensor using fluorescein as dye-transducer to convert the change in cofactor absorption observed during the reaction on D-Ser into a change in fluorescence intensity [86]. More recently, a noninvasive system was constructed using luminescent DNA/silver nanoclusters (DNA/Ag NCs) to detect D-Ala and D-Pro as biomarkers to diagnose gastric cancer, merging D-AA oxidation and the Fenton reaction [76]. The detection was completed in less than 1 h, and the LOD values (0.1 μM and 1 μM for D-Ala and D-Pro, respectively, Table 2) were suitable to assay the effective concentration of these D-AAs in gastric cancer at an early stage. 

A fluorescent biosensor based on aptamers and gold NPs was used to assay D-Arg levels [77]. Carboxyfluorescein-labeled aptamers were absorbed on Au-NPs and their fluorescence signal was quenched based on fluorescence resonance energy transfer. When D-Arg was added to the reaction mixture, the aptamer was released from Au-NPs, resulting in an enhanced fluorescence directly proportional to the D-AAs concentration, in the 0 to 300 nM range. The biosensor was successfully applied to human urine samples with satisfactory results; the maximum fluorescence signal was reached within 45 min and the value was stable within 2 h [77].

### 5.4. Alternative Biosensors

**Figure 5:**
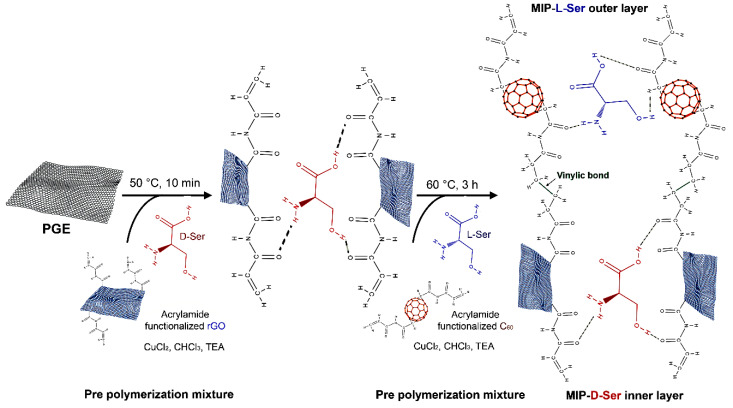
Scheme of the electrochemical biosensor to assay both D- and L-Ser [78]. The system was obtained by generating acrylamide functionalized reduced graphene oxide-fullerene, assembling dual molecularly imprinted polymers (MIPs) layer-by-layer: a pencil graphite electrode (PGE) was initially spin coated with D-Ser imprinted acrylamide functionalized reduced graphene oxide (rGO) and, after a thermal treatment, the electrode was again modified with L-Ser imprinted acrylamide functionalized fullerene molecules (C_60_). Modified from [78].

Since the level of a single D-AA in biological samples can significantly differ among individuals (because of differences in nutrition and in gut microbiota), the use of their concentration can be misleading: accordingly, the estimation of the D/(D+L)-AA ratio seems a more suitable parameter for the identification of pathological conditions [87,88,89]. A novel method to quantify both D- and L-Ser was recently obtained by generating acrylamide functionalized reduced graphene oxide-fullerene, assembling dual imprinted polymers layer-by-layer. In detail, a pencil graphite electrode was initially spin coated with D-Ser-imprinted acrylamide functionalized reduced graphene oxide, and, after a thermal treatment, the electrode was again modified with L-Ser-imprinted acrylamide functionalized fullerene molecules, Figure 5 [78]. By using this electrochemical biosensor, the two enantiomers could be sequentially analyzed at one electrode surface, with high sensitivity (LOD of 2.3 µM) in a wide concentration range of analytes (7–190 µM). It was used to quantitatively determine D-Ser and L-Ser in blood serum, cerebrospinal fluid, and pharmaceuticals.

## 6. Summary and Conclusions

Over the last 10 years, the field of biosensors has developed rapidly, mainly owing to the use of novel biorecognition molecules and nanomaterials and to better multidisciplinary collaboration between the life sciences and engineering [12]. The design of a biosensor for D-AAs must take into account of both the target analyte and the matrix composition. When the biosensor comes into contact with biological fluids, one of the most challenging drawbacks is the interference from fouling agents and chemicals present in the sample matrix, such as small metabolites, proteins, and macromolecules [90,91,92]. These problems can be overcome by pretreating samples using extraction, filtration, and derivatization methods [10,93]: these procedures are routinely employed for food samples but are not feasible when an online evaluation is required. The direct application of biosensors to (biological) samples is the most desirable set up. Accordingly, novel biosensors employed several polymeric films, such as *o*-phenylenediamine, and specific membranes, such as Nafion and hydrogels, as well as graphene-based composite materials [94,95].

In this context, irrespective of the type of biosensor, reducing the size to micro- or nanoscale resulted in a better signal-to-noise ratio and, taken together with the smaller sample volume used, assay costs were lower. For instance, optical biosensors coupled to quantum dots (i.e., semiconductor crystals with peculiar quantum confinement effects), CNTs, and microfluidic- and graphene-based biosensors, as well as lab-on-a-chip devices, are widely reported in the literature for electrode fabrication [12,17,96], and have been also applied for detecting D-AAs.

Applying micro- and nanobiosensors to SECM represents a valuable innovation in the field [84]; imaging surface topography and local reactivity analysis (Figure 4) make it possible to map the release of D-AAs (especially the neuromodulators D-Ser and D-Asp) from astrocytes, neurons, and tissues, giving further insight into the role of these biomolecules in brain development and function.

## 7. Future Perspectives

The production of a commercial biosensor must take into account market analysis, advantages over existing methods, stability of the device over the time, and costs [12]. Biosensors for detecting D-AAs can now be exploited in different sectors. Of course, researchers should further focus on generating simple and inexpensive online devices for food-processing analysis, especially trying to avoid the need to pretreat samples. In terms of sensitivity, present instrumentation provides the required sensitivity but lacks in selectivity in terms of ability to distinguish the different D-AAs present in a solution. Here, protein engineering of stereoselective enzyme variants active on a single D-AA [55,56] could generate a bioreceptor for such an application. 

The aforementioned system that is able to assay both L-Ser and D-Ser (Figure 5) also represents a relevant innovation in the field [78]. In fact, for many pathological conditions, the most reliable parameter is the D-/(D+L)-AA ratio, a value that allows a better comparison between different samples but that normally requires time-consuming and complicated HPLC chiral analyses [11,36,97,98,99]. 

Finally, implantable biosensors and surface-scanning biosensors will offer innovative tools for future research aimed at investigating the neurological role of D-AAs, a vibrant field that is growing at an accelerating pace. In past years, considerable progress in understanding D-AAs biology has been made, but the determination of these molecules constitutes a challenging task that can confound observations: we encourage the use of appropriate procedures with multiple controls, as recently suggested by [100].

## Figures and Tables

**Table 1:** Comparison of analytical properties of different electrochemical biosensors to detect D-enantiomers of amino acids (D-AAs) in foods. LOD: limit of detection; DAAO: D-amino acid oxidase.

DAAO Source	Optimal Temperature (°C)	Response Time	LOD (mM)	Detected D-AAs	Application	Ref.
*R. gracilis*	25	5 min	0.15	Ala	Milk	[21]
	25	15 min	0.25	Ala, Gln, Glu, Lys, Met	Grana Padano cheese	[22]
Pig kidney	40	1 min	0.2	Ala, His, Ile, Leu, Met, Phe, Pro, Trp, Val	Beer fermentation	[59]
	25	12 min	0.05	Ala	Fish sauce	[60]
	25	4 min	0.47	Ala, Arg, Met, Phe, Pro, Val	Milk	[61]
	25	3 min	0.001–0.03	Ala	Milk, fruit juice	[62]
	30	2 s	0.0002	Ala	Fruit juice	[63]
Goat kidney	35	3 s	0.001	Ala	Fruit juice	[64]

**Table 2:** Comparison of analytical properties of different biosensors to detect D-AAs for biomedical applications.

Bioreceptor	Assay Technique	Response Time	LOD (μM)	Biological Sample	Ref.
pkDAAO	Amperometric	6 min	60	Urine	[66]
	Amperometric	6 min	1000		[67]
	Amperometric	10 s	0.02	Serum	[68]
	Amperometric	2 min	6	*V. cholerae* cultures	[69]
	Amperometric	5 s	0.04	Serum	[70]
	Amperometric	50 s	2	Urine	[71]
DAAO from goat kidney	Amperometric	1 s	1.5	Serum, urine	[65]
RgDAAO	Amperometric	2 s	0.016	Rat frontal cortex	[72]
	Amperometric	4 s	0.008	Rat frontal cortex	[73]
	Amperometric	2 min	0.6	*Xenopus laevis* brain	[74]
	Amperometric	10 s	0.17	Rat brain	[75]
DNA	Fluorimetric	60 min	0.1-1	Saliva	[76]
Aptamer	Fluorimetric	45 min	0.002	Urine	[77]
Dual imprinted polymer	Amperometric	3 min	2.3	Serum, brain, drugs	[78]

## Abbreviations

AAs	α-amino acids
Au-NF	Gold nanofilm
CNTs	Carbon nanotubes
CuNPs	Copper nanoparticles
D-AAs	D-enantiomer of α-amino acids
DAAO	D-amino acid oxidase
DNA/Ag NCs	DNA/silver nanoclusters
FAD	Flavin adenine dinucleotide
FIA	Flow-injection analysis
L-AAs	L-enantiomer of α-amino acids
LOD	Limit of detection
LOQ	Limit of quantification
MIPs	Molecularly imprinted polymers
MWCNTs	Multiwall carbon nanotubes
NMDA	N-methyl-D-aspartate
PANI	Polyaniline
pkDAAO	D-amino acid oxidase from pig kidney
PGE	Pencil graphite electrode
PO	Pyruvate oxidase
PPD	Poly-*m*-phenylenediamine
PTh	Polythiophene
RgDAAO	D-amino acid oxidase from *Rhodotorula gracilis*
rGO	Reduced graphene oxide
SECM	Scanning electroanalytical technique
ZnSNPs	Zinc sulfide nanoparticles
